# Home Management of Children With COVID-19 in the Emilia-Romagna Region, Italy

**DOI:** 10.3389/fped.2020.575290

**Published:** 2020-10-23

**Authors:** Gianluca Vergine, Michela Fantini, Federico Marchetti, Marcello Stella, Enrico Valletta, Giacomo Biasucci, Marcello Lanari, Icilio Dodi, Maurizio Bigi, Anna Maria Magista, Francesca Vaienti, Andrea Cella, Paola Affanni, Maria Carla Re, Vittorio Sambri, Susanna Esposito, The Regione Emilia-Romagna COVID-19 Pediatric Working Group (RERCOPed)

**Affiliations:** ^1^Department of Pediatrics, Infermi Hospital Rimini, Azienda Sanitaria Locale Romagna, Rimini, Italy; ^2^Unit of Microbiology, The Great Romagna Area Hub Laboratory, Pievesestina di Cesena, Italy; ^3^Department of Pediatrics, Santa Maria delle Croci Hospital, Ravenna, Italy; ^4^Department of Pediatrics, Azienda Sanitaria Locale Romagna, Forlì, Italy; ^5^Pediatric Clinic, Azienda Sanitaria Locale Romagna, Cesena, Italy; ^6^Pediatrics and Neonatology Unit, Guglielmo da Saliceto Hospital, Piacenza, Italy; ^7^General and Emergency Pediatrics, Policlinico Sant'Orsola, University of Bologna, Bologna, Italy; ^8^General and Emergency Pediatrics, Pietro Barilla Children's Hospital, Parma, Italy; ^9^Pediatric Community Unit, Azienda Sanitaria Locale Romagna, Rimini, Italy; ^10^Pediatric Community Unit, Azienda Sanitaria Locale Romagna, Ravenna, Italy; ^11^Laboratory of Hygiene and Public Health, Department of Medicine and Surgery, University of Parma, Parma, Italy; ^12^Microbiology Unit, Policlinico Sant'Orsola, University of Bologna, Bologna, Italy; ^13^Pediatric Clinic, Pietro Barilla Children's Hospital, University of Parma, Parma, Italy

**Keywords:** community health service, COVID-19, home management, pediatric infectious diseases, SARS-COV-2

## Abstract

In most children, coronavirus disease 2019 (COVID-19) is a mild or moderate disease. Moreover, in a relevant number of cases, severe acute respiratory syndrome coronavirus 2 (SARS-CoV-2) infection remains totally asymptomatic. All these findings seem to suggest that otherwise healthy children with suspected COVID-19 might be managed in the community in most cases, thus avoiding hospital admission and closely related medical, social and economic problems, including overwhelming hospitals. Unfortunately, home management of children with suspected COVID-19 rarely occurs, and many children with suspected or laboratory-confirmed SARS-CoV-2 infection are frequently hospitalized irrespective of the severity of disease. To evaluate the role of community health houses (CHHs) in the management of children with COVID-19, 1,009 children with suspected SARS-CoV-2 infection were studied in Emilia-Romagna Region, Italy. Among them, 194 (19.2%) resulted positive for SARS-CoV-2. The majority (583, 58%) were tested at home by CHHs, while 426 (42%) were brought to the hospital for testing. The patients who were managed in the hospital had a significantly lower median age than those who were managed at home (2 vs. 12 years, *p* < 0.001). Exposure to SARS-CoV-2 cases within the family was significantly more frequent among those who were managed at home (82 vs. 46%, *p* < 0.05). The clinical findings were similar between the children who were managed at home and those who were managed in the hospital. Only one of the children managed at home (0.7%) required hospitalization; in comparison, 26 (48%) of those whose swab samples were taken at the hospital were hospitalized. Our research shows for the first time the importance of CHHs in the management of COVID-19 in children; because of the high frequency of mild to moderate cases, management by CHHs can reduce the care load in hospitals, providing enormous advantages on the familial, medical, social, and economic levels. These findings could be useful for suggesting a territorial rather than hospital-based strategy in pediatrics in the case of a new wave of the epidemic.

## Introduction

Coronavirus disease 2019 (COVID-19) is usually mild or moderate for most children (1, 2). Moreover, in a relevant number of cases, severe acute respiratory syndrome coronavirus 2 (SARS-CoV-2) infection remains totally asymptomatic (3). Severe cases requiring hospitalization or admission to the intensive care unit are rare and are expected in children with severe chronic underlying disease (4). Death is an exceptional event. When symptomatic, children with COVID-19 generally have signs and symptoms of a mild upper respiratory tract infection with low fever, cough, sore throat and fatigue. In 10% of cases, gastrointestinal manifestations, such as mild diarrhea and vomiting, are associated or can be the only signs and symptoms of disease (1, 5). All these findings seem to suggest that otherwise healthy children with suspected COVID-19 might be managed in the community in most cases, thus avoiding hospital admission and closely related medical, social and economic problems.

Unfortunately, home management of children with suspected COVID-19 rarely occurs, and many children with suspected or laboratory-confirmed SARS-CoV-2 infection are frequently hospitalized irrespective of the severity of disease. A recent survey carried out in Italy in 17 pediatric emergency departments showed that 38 out of 100 children with documented SARS-CoV-2 infection were hospitalized, even though only 4 patients had oxygen saturation levels below 95% and only 9 required respiratory support (5). Several factors can explain this finding, including poor knowledge among parents and health care workers regarding the characteristics of COVID-19 in children and the fear that children could develop a severe disease that would be difficult to control at home Community health houses (CHHs) can avoid admission to the hospital emergency department by identifying children with a reasonable suspicion of COVID-19, performing basal laboratory investigations to confirm the diagnosis in an outpatient setting, selecting subjects who need hospitalization and organizing home monitoring for those with mild COVID-19 who can return home. To evaluate this hypothesis, the management of children with COVID-19 in Emilia-Romagna, a region of northern Italy with long-established and well-functioning CHHs, was studied.

## Materials and Methods

The medical records of children and adolescents (<19 years old) admitted to hospital emergency departments or CHHs in the Emilia-Romagna region for suspected SARS-CoV-2 infection from March 1 to April 15, 2020 were retrospectively reviewed. In both settings, after each patient's data were deidentified, the reasons for suspicion of COVID-19, the measures used to confirm the diagnosis (including laboratory and radiological tests), the clinical manifestations, the management of disease and the characteristics of follow-up were retrieved and analyzed, taking into account the institution where each child had been initially treated. All of the institutions performed nasopharyngeal swabs for SARS-CoV-2 using a real-time reverse-transcriptase polymerase-chain-reaction assay, as previously reported (6). The study was approved by the ethics committees of the participating centers in April. Data collection was allowed by the written consent of both parents.

The groups were compared using the chi-squared or Kruskal-Wallis test, when appropriate. All of the analyses were two tailed, and *p*-values <0.05 were considered statistically significant. All analyses were conducted using Stata/SE 14.2 (College Station, TX: StataCorp LLC, USA). Multivariate logistic regression analysis was performed to evaluate variables which may be associated with hospitalization in positive patients.

## Results

Overall, in the study period, 1,009 children and adolescents with suspected SARS-CoV-2 infection (548, 54.3% males; median age, 9.5 years) were evaluated in the Emilia-Romagna Region, Italy. Among them, 194 (19.2%) resulted positive for SARS-CoV-2. Their demographic characteristics are reported in Table 1. The majority (583, 58%) were tested at home by CHHs' services, while 426 (42%) were brought to the hospital for testing (35% of them waited for the results in the emergency room, and 65% waited for the results at home). Age and gender distribution were similar between the SARS-CoV-2-positive and negative cases. Exposure to SARS-CoV-2 cases was reported and confirmed in 48% of the cases and was significantly more frequent in the SARS-CoV-2-positive cases than in the SARS-CoV-2-negative cases (*p* < 0.05). The most common reason for performing the swab test was evidence of signs and symptoms of respiratory infection in the presence of known contact with a COVID-19-positive adult. A minority of the children were tested as asymptomatic contacts of positive household members or for screening before a previously scheduled surgery, and there was no significant difference between the SARS-CoV-2-positive cases and SARS-CoV-2-negative cases.

**Table 1:** Characteristics of children with suspected severe acute respiratory syndrome coronavirus 2 (SARS-CoV-2) infection in the Emilia-Romagna Region, Italy.

**Characteristics**	**Overall number of cases (*n* = 1,009)**	**Overall SARS-CoV-2 positive cases (*n* = 194)**	**Overall SARS-CoV-2 negative cases (*n* = 815)**
Median age (range)—years	9.5 (0–19)	11 (0–18)	9 (0–19)
Age distribution, no. (%)			
<1 year	101 (10%)	24 (12%)	77 (9%)
1–5 years	252 (25%)	29 (15%)	223 (27%)
6–10 years	214 (21%)	37 (19%)	177 (23%)
>10 years	442 (44%)	104 (54%)	338 (41%)
Sex, no. (%)			
Male	548 (54%)	108 (56%)	440 (52%)
Female	461 (46%)	86 (44%)	375 (46%)
Known exposure to SARS-CoV-2, no. (%)	482 (48%)	141 (73%)	340 (42%)
Family cluster	402 (50%)	139 (98%)*	263 (77%)
Other exposure	80 (10%)	3 (2%)	77 (13%)
Indication for swab, no. (%)	701 (69%)	160 (82%)	541(66%)
Symptoms and contact	436 (62%)	117 (73%)	319 (59%)
Symptoms without contact	201 (29%)	23 (15%)	178 (33%)
Asymptomatic contact	38 (5%)	18 (11%)	20 (4%)
Screening for surgery	26 (4%)	2 (1%)	24 (4%)
Location where swab was performed			
Hospital	426 (42%)	54 (28%)	372 (46%)
Home	583 (58%)	140 (72%)	443 (54%)

*Comparison was made between SARS-CoV-2 positive cases and the SARS-CoV-2 negative ones*.

* *p < 0.05 vs. overall SARS-CoV-2 negative patients; no other significant difference between the groups*.

Table 2 summarizes the characteristics of the children who were positive for SARS-COV-2 according to the management setting. Overall, in 140 of 194 patients (72.2%), the swab sample was taken at home, whereas in 54 patients the swab sample was taken in the hospital. The patients who were managed in the hospital had a significantly lower median age than those who were managed at home (2 vs. 12 years, *p* < 0.001). Family exposure was significantly more frequent among those who were managed at home (82 vs. 46%, *p* < 0.05). The clinical findings were similar between the children who were managed at home and those who were managed in the hospital, with a large proportion of the patients presenting with fever. None of the children presented with respiratory failure, and median duration of hospitalization was of 2.5 days (range, 1–6 days). Chest radiographs and blood exams were performed significantly more often for the hospital patients than for those who were managed at home (20 and 44% vs. 1 and 3%, respectively, *p* < 0.05). Paracetamol alone was the main therapy for the patients who were managed at home (89 vs. 37.5% among those managed in the hospital, *p* < 0.05); in contrast, antibiotics were used by half of the hospitalized children (50 vs. 0% among those managed at home, *p* < 0.05). Only one of the children managed at home (0.7%) required hospitalization; in comparison, 26 (48%) of those whose swab samples were taken at the hospital were hospitalized.

**Table 2:** Characteristics of children who tested positive for severe acute respiratory syndrome coronavirus 2 (SARS-CoV-2) according to management setting in the Emilia-Romagna Region, Italy.

**Characteristics**	**SARS-CoV-2 positive children with swab performed at home (*n* = 140)**	**SARS-CoV-2 positive children with swab performed at the hospital (*n* = 54)**
Median age (range)—years	12 (0–18)^∧^	2 (0–18)
Age distribution, no. (%)		
<1 year	5 (3%)^∧^	19 (35%)
1–5 years	14 (10%)	15 (28%)
6–10 years	32 (23%)	5 (9%)
>10 years	89 (64%)*	15 (28%)
Sex, no. (%)		
Male	71 (51%)	37 (69%)
Female	67 (49%)	17 (31%)
Exposure to SARS-CoV-2, no. (%)		
Family cluster	114 (82%)*	25 (46%)
Other exposure	2 (1%)	1 (2%)
Indication for swab, no. (%)	121	39
Symptoms	105 (86%)	35 (90%)
Asymptomatic contact	16 (14%)	2 (5%)
Screening for surgery	0 (0%)	2 (5%)
Symptoms, no. (%)		
Fever	90 (71%)	41 (89%)
Cough	34 (27%)	23 (50%)
Shortness of breath	1 (0%)	3 (6.5%)
Conjunctivitis	13 (10%)	1 (2%)
Rhinorrhea	26 (20%)	8 (17%)
Sore throat	11 (9%)	12 (26%)
Diarrhea	9 (7%)	10 (22%)
Requested exams, no. (%)		
Chest radiograph	2 (1%)*	11 (20%)
Chest CT	2 (1%)	1 (2%)
Blood exams	4 (3%)^∧^	24 (44%)
Therapy, no. (%)	18 (13%)*	24 (24%)
Paracetamol only	16 (89%)*	9 (37.5%)
Antibiotics	1 (5.5%)*	12 (50%)
Hydroxychloroquine	1 (5.5%)	0 (0%)
Outcome, no. (%)		
Hospitalized	1 (0.7%)^∧^	26 (48%)
Admitted to ICU	0 (0%)	1 (2%)
Survived	140 (100%)	54 (100%)

*Comparison was made between SARS-CoV-2 positive children with swab performed at home and the SARS-CoV-2 positive ones with swab performed at the hospital*.

* *p < 0.05 and ^∧^p < 0.001 vs. SARS-CoV-2 positive children managed at the hospital; no other significant difference between the groups*.

*ICU, intensive care unit*.

Children under 1 year of age who were SARS-CoV-2 positive were hospitalized and received antibiotics in a significantly higher proportion than those who were older (50 vs. 17% in children 1–5 years old, 8% in those 6–10 years and 7% in those 10–19 years; *p* < 0.05). No other significant difference was observed between the groups.

Table 3 shows univariate and multivariate logistic regression analysis. In multivariate analysis hospitalization was associated with the swab being performed in the hospital itself (odds ratio: 64.46, 95% confidence intervals: 6.56–633.10).

**Table 3:** Univariate and multivariate logistic regression analysis showing association between different factors and hospitalization in SARS-CoV-2 positive children and adolescents.

	**Univariate**		**Multivariate**	
**Hospitalization**	**OR (95% CI)**	***p*-value**	**OR (95% CI)**	***p*-value**
Age, years				
<1				
1–5	0.21 (0.06–0.73)	0.014	0.09 (0.01–0.89)	0.040
6–10	0.09 (0.02–0.37)	0.001	0.26 (0.21–3.17)	0.292
>10	0.07 (0.02–0.21)	0.000	0.17 (0.02–1.34)	0.093
Male gender	0.58 (0.25–1.38)	0.219	0.99 (0.20–4.94)	0.993
Family exposure	0.46 (0.24–0.892)	0.021	0.50 (0.09–2.53)	0.405
Location of test in hospital yes/no	129.1 (16.8–990)	<0.0001	64.46 (6.56–633.10)	<0.0001
Fever	3.12 (0.39–24.7)	0.280	6.76 (0.48–93.54)	0.154
Therapy: yes/no	5.35 (2.27–12.6)	<0.0001	2.30 (0.42–12.68)	0.336

*95% CI, confidence intervals; OR, odds ratio*.

## Discussion

This study shows that children with COVID-19 can be successfully managed at home, at least in places where CHHs are available and well-functioning. Almost all of the children were followed at home, and those with positive swabs for SARS-CoV-2 were managed without relevant clinical problems. Moreover, this study confirms what has already been reported in all epidemiological evaluations of COVID-19 in pediatric patients, i.e., that children generally have mild disease or are asymptomatic, and in most cases, the emergence of respiratory signs and symptoms of a mild upper respiratory infection is the main reason for requesting a medical evaluation during the COVID-19 pandemic (7).

The availability in the community of CHHs that are able to perform nasopharyngeal swabs for SARS-CoV-2 identification, send samples to the laboratory, collect results and organize adequate home monitoring of SARS-CoV-2 infected children may significantly reduce the overwhelming of hospitals, which has generally been very common worldwide during the pandemic. In this study, only one child among those diagnosed in the community needed hospitalization after he returned home, compared to ~50% of those admitted to the emergency department of the hospital. Furthermore, hospitalized patients had a significantly higher number of laboratory tests and chest radiographs, which seemed only partially justified by the clinical characteristics of COVID-19 in the two groups of children.

The signs and symptoms of the disease were similar in the patients managed at home and in those who were hospitalized, although the prevalence of fever, cough and shortness of breath was slightly higher in the hospitalized children than in those followed at home. Moreover, only one of the hospitalized children needed admission to the intensive care unit, suggesting that even in hospitalized children, COVID-19 is a mild or moderate disease. Finally, hospitalization might be linked more to assessments of the potential danger of COVID-19 than to real clinical needs. The hospitalized children were significantly younger than those managed at home, and they might have been hospitalized out of fear of a potential negative evolution of COVID-19 in younger children than for real clinical reasons. On the other hand, studies have highlighted that among children with severe COVID-19, those with the highest risk of developing severe clinical manifestations are the youngest, particularly those younger than 5 years (8–11).

Limitations of these study include its retrospective design and the fact that it was conducted in a single Region of Italy. However, we did not have missing data and this represents a strength point of our research. Moreover, Emilia-Romagna, that was one of the Italian Regions with the greatest COVID-19 incidence and the highest numbers of disease-related deaths, has a population <18 years of 777,933 subjects and this study reported data from 6 out of 9 provinces where more than 80% of the SARS-CoV-2 positive children and adolescents were diagnosed and followed. In addition, Emilia-Romagna is the Italian Region with the longest history of CHHs, and it is difficult to find other Italian Regions with a similar established territorial medical organization. In our research, information coming from 85 out of 107 (79.4%) CHHs is reported and for this reason we think that our findings are really representative of a well-functioning healthcare model. On the other hand, our multivariate analysis confirmed that hospitalization is not related to disease's severity but to where swabs for SARS-CoV-2 were performed: in the hospital or not.

In conclusion, although further studies are needed, our research shows for the first time the importance of CHHs in the management of COVID-19 in children; because of the high frequency of mild to moderate cases, management by CHHs can reduce the care load in hospitals, providing enormous advantages on the familial, medical, social, and economic levels. These findings could be useful for suggesting a territorial rather than hospital-based strategy in pediatrics in the case of a new wave of the epidemic and highlight the need of strict criteria for hospitalization in SARS-CoV-2 positive children.

## Data Availability Statement

The raw data supporting the conclusions of this article will be made available by the authors, without undue reservation.

## Ethics Statement

The studies involving human participants were reviewed and approved by Ethics Committee of Azienda Sanitaria Locale Romagna and Emilia-Romagna Region. Written informed consent to participate in this study was provided by the participants' legal guardian/next of kin.

## Author Contributions

GV designed the study, enrolled the patients, and co-wrote the first draft of the manuscript. MF was the study coordinator and performed the statistical analysis. FM, MS, EV, GB, ML, ID, MB, AM, FV, and AC enrolled the patients and gave a substantial scientific contribution. PA, MR, and VS performed the laboratory analyses. SE wrote the first draft of the manuscript and made substantial scientific contributions. All authors approved the final version of the manuscript.

## Conflict of Interest

The authors declare that the research was conducted in the absence of any commercial or financial relationships that could be construed as a potential conflict of interest.
